# Case of Croup

**Published:** 1823-04

**Authors:** H. S. Belcombe


ART. VII.—*Case of Croup.* By H. S. BELCOMBE, M.D.

DECEMBER 5, 1822, 3 o'clock, P.M. Master C. a fine healthy boy, ten years old, was suddenly seized, after eating a hearty dinner, with cough, difficulty of breathing, and sense of suffocation. I had the good fortune to see him in less than an hour from the commencement of the attack, and found him labouring under all the symptoms of croup; the brazen sound more particularly marked than I ever recollect to have heard. Three grains of tartarized antimony, dissolved in two ounces of water, and given in the course of twenty minutes, having procured copious vomiting, fourteen leeches were applied to the throat, and the bleeding well promoted by warm poultices. In five hours, there being no remission of the symptoms, my friend, Mr. Wood, who met me in consultation, opened a vein, and abstracted ten ounces of blood, dark and thick, (but which threw up no inflammatory crust, on cooling:) this seemed to alleviate his misery; and three grains of calomel were ordered every two hours through the night, with a blister to the sternum.

In the morning, we found he had passed a tranquil night. The cough was yet hard, with sense of constriction about the glottis; the pulse sharp and hurried, but much less so than in the evening. Fourteen leeches were again applied to the throat; an aperient draught ordered, and the calomel continued as before.

On the morning of the 7th, the inflammatory symptoms had yielded, and the calomel was, in consequence, discontinued. A cough still remained, but it was loose, and unattended with pain. Some bilious fetid motions had passed, but the boiled-spinage appearance was totally wanting. The boy is becoming convalescent, and does not appear to suffer any inconvenience from the quantity of calomel exhibited.

I have no doubt that, in this case, the bleeding and calomel arrested the inflammatory symptoms, and probably saved the boy's life; and it may seem a gratuitous observation to say I would recommend the same practice in all possible cases; but there does appear to me, in the practice of medicine at this day, a fashion of depending upon some one particular remedy, instead of conjoining all the powers of the healing art,—and this, especially in inflammatory diseases, is earnestly to be deprecated. The publication, also, of a celebrated northern Professor, on the Use and Abuse of Mercury, may have tended to injure the fame of that potent remedy. Loth as I am to differ from so respectable an authority, I must say, that, after an attentive perusal of his work, and a thoughtful investigation of the results of my own practice, (for I am not bold enough to say that a cure is always to be considered as the criterion of the efficacy of a medicine,) I cannot agree with him in his opinions. This is a subject, however, to lead too far at present, and I am unwilling to trespass upon your pages.

*Newcastle, Staffordshire; Dec.* 10, 1822.

